# Noninvasive Biomarkers for Early Detection of Primary Sclerosing Cholangitis in Ulcerative Colitis

**DOI:** 10.1155/bmri/9547868

**Published:** 2026-10-17

**Authors:** Pedram Asadi Sarabi, Alireza Beheshti-Maal, Amir Ali Judaki, Seyedeh Kiana Teymoorian, Zohreh Hashemian, Elham Rismani, Asma Gul, Reza Malekzadeh, Moustapha Hassan, Massoud Vosough

**Affiliations:** ^1^ Department of Regenerative Medicine Cell Science Research Center Royan Institute for Stem Cell Biology and Technology ACECR Tehran Iran acecr.ac.ir; ^2^ Faculty of Sciences and Advanced Technologies in Biology University of Science and Culture Tehran Iran usc.ac.ir; ^3^ Molecular Medicine Department Biotechnology Research Center (BRC) Pasteur Institute of Iran Tehran Iran pasteur.ac.ir; ^4^ Department of Biological Sciences Faculty of Sciences International Islamic University Islamabad Pakistan iium.edu.my; ^5^ Digestive Disease Research Institute Tehran University of Medical Sciences Tehran Iran tums.ac.ir; ^6^ Experimental Cancer Medicine Institution for Laboratory Medicine Karolinska Institute Stockholm Sweden ki.se

**Keywords:** S100A8, S100A12, gene expression profiling, primary sclerosing cholangitis, ulcerative colitis

## Abstract

Primary sclerosing cholangitis (PSC) is a progressive cholestatic liver disease strongly associated with ulcerative colitis (UC). Although uncommon, PSC‐associated ulcerative colitis (PSCUC) represents a distinct subtype of inflammatory bowel diseases characterized by a higher risk of colorectal and hepatobiliary cancers. Early diagnosis remains a challenging issue because of the lack of specific biomarkers. Bioinformatic analysis of Gene Expression Omnibus (GEO) datasets identified potential, specific, and noninvasive biomarkers via differential gene expression and functional enrichment. After processing the data, *S100A8* and *S100A12* were identified as candidate biomarkers for detecting PSC in patients with UC. *S100A8* encodes one component of calprotectin, the S100A8/S100A9 heterodimer widely used as a marker of intestinal inflammation. These two genes are primarily involved in inflammation, immune responses, and leukocyte recruitment, making them potentially useful biomarkers for early detection in PSC in UC patients. These biomarkers offer potential implications for disease stratification and therapeutic targeting. Further validation in prospective clinical studies is warranted to confirm their diagnostic and prognostic utilities.

## 1. Introduction

Primary sclerosing cholangitis (PSC) is defined as a chronic and progressive liver disorder, in which inflammation and scars in the bile ducts can lead to liver failure, cholestasis, and increase the risk of various cancers in humans [1]. Also, PSC is known for fibrosis, inflammation, and structuring of intrahepatic and/or extrahepatic bile ducts. Although PSC remains relatively uncommon (~40 per 100,000), its burden is rising, and the clinical stakes are high. Genetic predisposition is a significant factor in PSC. HLA and non‐HLA susceptibility loci, as well as immune processes, are recognized as key risk factors for PSC. However, the exact etiology of this disease is still unclear [2]. PSC is most commonly diagnosed between 30 and 40 years of age. Unlike most autoimmune disorders, PSC is more prevalent in the male population, comprising 65%–70% of the patient pool [3]. About 70% of patients with PSC have ulcerative colitis (UC) [4]. Contrastingly, approximately 5% of patients with UC have concomitant PSC [5]. UC is the most common form of inflammatory bowel disease (IBD), which is characterized by ulcers on the lining of the colon and rectum [6]. Although liver biopsy provides significant information about PSC, such as fibrosis stage, noninvasive methods are essential and are preferred over invasive methods. Early detection of PSC in patients with UC is critical because of its progressive nature, risk of cholestatic liver failure, and increased incidence of colorectal and hepatobiliary malignancies [7]. Despite its low prevalence, PSC remains a significant concern in UC patients, often developing silently before clinical symptoms arise. Magnetic resonance cholangiopancreatography (MRCP) is the gold standard for diagnosing PSC, providing a noninvasive assessment of bile duct abnormalities [8]. However, in cases of small‐duct PSC, where MRCP findings are inconclusive, liver biopsy remains a diagnostic option [9]. Using specific blood biomarkers to indicate PSC in patients with UC can be a valuable, safe method for early diagnosis. Earlier diagnosis of PSC in UC patients (PSCUC) can enhance treatment strategies and disease progression monitoring [10].

Bioinformatics and in silico studies are tools that can provide insights into biomarker discovery. This powerful approach analyzes large‐scale, complex omics datasets, including genomics, proteomics, metabolomics, and transcriptomics [11, 12]. In this study, two RNA sequencing datasets were selected to identify blood‐based biomarkers for PSC patients with UC. RNA sequencing profiles of these studies were analyzed to determine the most specific biomarker for diagnosis. Gene enrichment analysis, gene expression, protein‐protein interactions, and gene ontology (GO) were carried out to gain insights into the target biomarker. After processing the data, *S100A8* and *S100A12* were identified as candidate biomarkers for detecting PSC in patients with UC. The S100A8/S100A9 heterodimer, known as calprotectin, is widely used in fecal testing as a noninvasive marker of intestinal inflammation in patients with IBD, whereas *S100A12* is a related but distinct S100‐family inflammatory protein [13, 14]. Selected biomarkers can aid early detection, serve as a safe diagnostic tool, and personalize treatment in clinical settings.

## 2. Methods

### 2.1. Data Sources and Sample Selection

Two publicly available RNA sequencing datasets, GSE177044 and GSE83687, were obtained from the Gene Expression Omnibus repository. In GSE177044, the primary UC comparison included 481 UC samples and 307 (non‐IBD) healthy‐control samples. The PSC‐alone comparison included 43 PSC‐alone samples and 43 selected (non‐IBD) healthy‐control samples, whereas the PSC/UC comparison included 173 PSC/UC samples and 173 selected (non‐IBD) healthy‐control samples. Samples were assigned to the respective case and control groups according to the diagnostic information provided in the GEO metadata. Non‐IBD control samples were selected from samples annotated as healthy controls in GEO2R, and no demographic or clinical filtering was applied. Age, sex, treatment status, disease duration, and other demographic or clinical characteristics were not used as selection criteria. Therefore, no individual‐level or frequency matching was performed, and these groups should be described as selected (non‐IBD) healthy‐control groups rather than matched controls.

For the GSE177044 whole‐blood dataset, eligibility required an unambiguous diagnosis of primary UC, PSC without concurrent UC, or PSC with concurrent UC, an age at disease onset of at least 13 years, and an age of at least 18 years at the time of blood sampling. Treatment status and disease duration were not considered eligibility criteria. As part of the original study′s sequencing quality‐control procedure, only RNA isolates with an RNA integrity number greater than 6 were included.

For the GSE83687 intestinal‐tissue dataset, UC specimens were obtained from patients undergoing bowel resection and were collected from intestinal regions showing moderate‐to‐severe inflammation. The diagnosis and pathological characteristics of each specimen were documented by the Mount Sinai Hospital Pathology Department. Non‐IBD control specimens consisted of histologically normal, noninflamed intestinal tissues collected more than 10 cm from the tumor in patients undergoing bowel resection for sporadic colon cancer. As part of the original study′s sequencing quality‐control procedure, only specimens with an RNA integrity number greater than 7 were included. For the present UC‐focused analysis, Crohn′s disease specimens and samples outside the predefined UC and non‐IBD control groups were excluded. No additional patient‐level eligibility criteria were applied in the present study beyond selecting the relevant diagnostic groups and available quality‐controlled expression profiles.

Available demographic and clinical characteristics were extracted from the publicly available GEO metadata and are summarized at the source‐cohort level in Table S1. Continuous variables were summarized as mean ± standard deviation and median with interquartile range, whereas categorical variables were summarized as counts and percentages among samples with available information. Missing values were not imputed. Because the accession identifiers of all selected GSE177044 control samples were not retained, demographic and clinical characteristics could not be reconstructed specifically for the selected analytical subsets.

### 2.2. Differential Gene Expression Analysis

Differential gene expression was analyzed using GEO2R in four disease–control comparisons: 481 primary UC samples versus 307 selected healthy‐control samples in whole blood, 43 PSC‐alone samples versus 43 selected (non‐IBD) healthy‐control samples in whole blood, 173 PSC/UC samples versus 173 selected healthy‐control samples in whole blood, and 32 UC intestinal‐tissue samples versus 60 non‐IBD control intestinal‐tissue samples. Samples were assigned to the respective case and control groups according to the diagnostic or clinical‐condition information provided in the GEO metadata and the sample‐selection procedure described in Section 2.1. No demographic or clinical matching variables were used to define the comparisons. Genes with an absolute log2 fold change ≥ 1 and a *p* − value of < 0.05 were considered differentially expressed.

To further assess whether the candidate genes were associated with PSC rather than reflecting general inflammatory activity in UC, additional direct disease‐disease comparisons were performed using GSE177044. These analyses included 173 PSC/UC samples versus 173 UC samples, 43 PSC‐alone samples versus 43 UC samples, and 43 PSC/UC samples versus 43 PSC‐alone samples. For the direct disease–disease comparisons involving UC, the UC samples were selected from samples annotated as primary UC in GEO2R, without applying demographic or clinical filtering. The accession identifiers of the selected UC subsets used in these direct comparisons were not retained; therefore, subset‐specific demographic characteristics could not be reconstructed. Differential expression was evaluated using GEO2R, and log2 fold change, nominal *p* − values, and Benjamini–Hochberg‐adjusted *p* − values were obtained for S100A8 and S100A12. An adjusted *p* − value < 0.05 was considered statistically significant. Because the UC‐only and PSC‐related samples originated from different source cohorts within GSE177044, comparisons involving UC were considered exploratory and potential cohort‐related effects were taken into account when interpreting the results.

### 2.3. Group‐Specific GO and KEGG Pathway Enrichment Analysis

To gain deeper insights into the functional implications of DEGs, we conducted GO enrichment analysis separately for each group, UC, PSC, and PSCUC in blood, and UC in tissue samples. The study was performed for both upregulated and downregulated genes in each group using the DAVID database (https://davidbioinformatics.nih.gov/). For each group, we evaluated enrichment across the three main GO categories: biological process (BP), cellular component (CC), and molecular function (mf). In addition, KEGG pathway enrichment analysis was performed to identify the biological pathways associated with the upregulated and downregulated genes in each condition. These group‐specific analyses allowed us to uncover unique and overlapping functional roles and pathways for the genes across the studied conditions, further enhancing our understanding of disease‐specific mechanisms.

### 2.4. Venn Diagram Analysis for Identification of Shared Upregulated Genes

To identify genes consistently upregulated across the selected disease‐control comparisons, Venn diagram analysis was performed using the differentially expressed gene lists obtained from four comparisons: primary UC versus selected healthy controls in whole blood, PSC alone versus selected healthy controls in whole blood, PSC/UC versus selected healthy controls in whole blood, and UC intestinal tissue versus non‐IBD control intestinal tissue. Only genes meeting the predefined differential‐expression criteria of a log2 fold change ≥ 1 and a *p* − value < 0.05 were included. Genes shared across all four comparisons were retained for subsequent extracellular‐space annotation and candidate‐biomarker prioritization. The analysis focused on upregulated genes to prioritize candidates showing increased expression in disease samples; however, gene overlap alone was not interpreted as evidence of disease specificity.

### 2.5. Identification of Extracellular Space Genes

We utilized the DAVID database to analyze the common genes identified across our four groups in the Venn diagram (https://davidbioinformatics.nih.gov/). We focused on extracellular space genes, which are more likely to be detected in blood samples. This is because extracellular space genes encode proteins secreted or localized outside the cell, making them accessible in the bloodstream. Identifying such genes increases the potential for identifying noninvasive biomarkers for disease diagnosis and monitoring

### 2.6. ROC Curve Analysis of Candidate Genes

ROC curve analyses were performed in Python using scikit‐learn to evaluate the discriminatory performance of *S100A8*, *S100A12*, and *IGHG3*. Disease samples were coded as one and controls as 0, and each gene was evaluated individually using its expression values. AUC values were calculated using roc_auc_score, and ROC curves were generated using roc_curve. No multivariable model or cross‐validation was applied.

### 2.7. Comparative Analysis of Differential Gene Expression Across Disease Conditions

To evaluate gene expression differences across multiple conditions, we conducted a comparative analysis of four groups: PSC, PSCUC, UC patients (whole blood), and UC patients (tissue). DEG was assessed based on log_2_ fold change for selected candidate genes. A scatter plot was generated to visualize and compare the log_2_ fold change of genes of interest across these conditions. This approach facilitated the identification of genes with consistent or divergent expression patterns between groups, contributing to biomarker discovery and disease‐specific profiling.

### 2.8. miRNA Networks Regulating Candidate Genes

We constructed an mRNA‐miRNA interaction network using the miRNet database (https://www.mirnet.ca/) to identify miRNAs associated with our candidate genes. This analysis focused on miRNAs involved in various diseases, particularly emphasizing their potential role in the pathogenesis of PSC and UC.

### 2.9. Coexpression Network Analysis

To explore the coexpression of PSC and UC genes with our candidate genes, we retrieved NCBI‐indexed PSC and UC genes using filters for Homo sapiens and protein‐coding genes. The common genes between PSC and UC were identified and combined with our candidate genes for network analysis. The coexpression network was constructed in Cytoscape using the ClusterViz algorithm [15]. Parameters were set with a clique size threshold of three and a complex size threshold of two. The network was analyzed to identify modules, focusing on modularity, node connectivity, and key clusters to uncover potential interactions and coexpression patterns between the genes. This approach facilitated the identification of biologically relevant gene clusters, including those containing key candidate genes.

### 2.10. Gene‐Drug Interaction Analysis

We analyzed the gene‐drug interactions for our candidate genes to explore potential therapeutic targets using the DGIdb database (https://dgidb.org/). This analysis allowed us to identify drugs and compounds that regulate or interact with the candidate genes. The interactions were visualized by constructing a gene‐drug interaction network, highlighting the drugs associated with each candidate gene. This approach provided insights into the regulatory potential of existing drugs and identified potential therapeutic agents relevant to PSC and UC pathogenesis.

### 2.11. Processing and Quality Control of Single‐Cell RNA Sequencing (scRNA‐seq) Datasets

scRNA‐seq datasets were obtained from the GEO under accession numbers GSE247128 and GSE231993. The final dataset comprised four standard colon samples, seven UC samples, three PSC samples, and two PSC samples with prior UC. Each sample was imported into R (v4.4.3) using the Seurat package (v5) [16]. Seurat objects were created for each sample, retaining cells that expressed at least three genes and genes detected in at least three cells. The percentage of mitochondrial gene content (“percent.mt”) was calculated by identifying genes with names starting with “MT‐”. Cells with > 20% mitochondrial content were excluded. Remaining cells were further filtered based on a median ± 2 standard deviations range for the number of detected features (“nFeature_RNA”) to remove outlier cells with unusual counts.

### 2.12. Normalization and Integration of scRNA‐seq Datasets

Following the standard Seurat v5.0 workflow [16] all samples were log‐normalized using the NormalizeData function with the LogNormalize method. Highly variable features were identified using the FindVariableFeatures function (selection.method = “vst”), with the feature limit set to “Inf” to retain all variable genes initially. Each sample was then scaled and centered using the ScaleData function. Dimensionality reduction was performed using Principal component analysis (PCA) via the RunPCA function in Seurat, which implements the Singular Value Decomposition (SVD) algorithm. Integration across datasets was achieved using Seurat′s reciprocal PCA (rPCA) integration algorithm, based on the top 10,000 variable features. The integrated objects were subsequently scaled, reclustered using Shared Nearest Neighbor (SNN) modularity optimization (via the FindClusters function, algorithm =  ^“^Louvain^”^), and visualized using the Uniform Manifold Approximation and Projection (UMAP) dimensionality reduction algorithm [17]. This combined computational pipeline ensured robust integration, noise reduction, and visualization of cell populations across the four biological conditions normal colon, UC, PSC, and PSC with UC.

### 2.13. Cluster Marker Identification and Annotation Using CellMarker 2.0

Markers for each cluster within all samples were identified using “FindAllMarkers” with a minimum log_2_ fold change of 1 and a *p* − value < 0.05, determined by the Wilcoxon rank‐sum test. The top annotated all clusters identified markers within each cluster using the CellMarker 2.0 database (https://www.bio-bigdata.center/).

### 2.14. Identification, Sub‐Setting, and Normalization of Monocyte Clusters

Within each integrated object, monocyte clusters were identified using cluster annotation and known markers, and were subset separately for each condition. All monocyte subsets were merged into a new Seurat object, with cell IDs prefixed to preserve their origin. Because of varying monocyte counts across conditions, a harmonization step was introduced. The count of cells expressing a candidate marker was normalized to a hypothetical population of 100,000 monocytes using the formula:

X=100,000×CM+/TCM.

Where:

• *X* = hypothetical population of candidate marker − positive cells.
• *C* *M* + = count of cells expressing the candidate marker.
• *T* *C* *M* = total count of monocytes.

This normalization allowed for comparing marker‐positive cell proportions across different sample sizes.

### 2.15. Visualization of *S100A8* and *S100A12* Expression and Harmonized Cell Counts

The expressions of *S100A8* and *S100A12* were visualized using Seurat′s “FeaturePlot” and “RidgePlot” functions. Additionally, the harmonized cell counts were plotted in scatter or dot plots to highlight differences across the four groups.

## 3. Results

### 3.1. Differential Gene Expression Analysis

The GEO2R differential expression analysis of GSE177044 and GSE83687 identified distinct gene expression profiles across the studied groups. For the analysis, we considered genes with a *p* − value less than 0.05 and a log_2_ fold change greater than or less than ± 1 as significantly differentially expressed. One hundred twenty‐seven genes were upregulated and 25 downregulated in the blood of UC patients. In PSC patients, 1577 genes were upregulated and 907 were downregulated. The PSCUC group in blood revealed 1283 upregulated and 820 downregulated genes. Additionally, 1233 genes were upregulated and 1138 downregulated in UC patients′ tissues. These findings highlight the unique and overlapping gene expression patterns across the different conditions and sample types (Table 1).

**Table 1:** Differential gene expression.

Group	Upregulated genes	Downregulated genes
UC (blood)	127	25
PSC (blood)	1577	907
PSCUC (blood)	1283	820
UC (tissue)	1233	1138

### 3.2. Shared and Distinct Pathway Enrichment in UC, PSC, and PSCUC

The enrichment analysis of DEGs across UC, PSC, and PSCUC (overlap of PSC and UC) in both blood and tissue samples revealed both distinct and overlapping BPs and pathways. In PSC‐blood, key enrichments included regulation of transcription by RNA polymerase II, the nucleus, protein binding, and pathways related to Alzheimer′s disease. PSCUC‐blood showed enrichment in positive regulation of transcription by RNA polymerase II, the nucleus, protein binding, and cancer‐related pathways, indicating overlaps with PSC in transcriptional regulation and CCs. In UC blood, processes such as the adaptive immune response, the extracellular region, antigen binding, and transcriptional dysregulation in cancer were prominent. UC‐tissue demonstrated enrichment in signal transduction, plasma membrane, calcium ion binding, and metabolic pathways, with some overlap with PSCUC in these pathways. Across these groups, transcription regulation, protein binding, and immune response processes are notably recurrent, highlighting shared molecular mechanisms while also underscoring disease‐specific differences in BPs and pathways (Figure 1).

**Figure 1:**
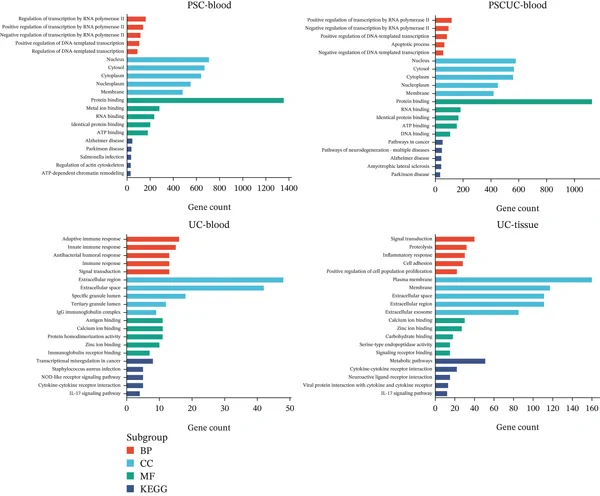
Gene Ontology (GO) and KEGG pathway enrichment analysis for PSC‐blood, PSCUC‐blood, UC‐blood, and UC‐tissue. Enrichment categories include biological processes (BP), cellular components (CC), molecular functions (MF), and KEGG pathways, highlighting the distribution of gene counts across key functional and pathway annotations.

### 3.3. Shared Genes Identified Through Venn Diagram Analysis

Venn diagram analysis of upregulated genes across four comparisons, primary UC versus selected healthy controls in whole blood, PSC alone versus selected healthy controls in whole blood, PSC/UC versus selected healthy controls in whole blood, and UC intestinal tissue versus non‐IBD control intestinal tissue identified five shared genes: *S100A12*, *S100A8*, *IGHG3*, *NEB*, and *CLEC4D*. These genes were retained for subsequent extracellular‐space annotation and candidate‐biomarker prioritization (Figure 2a).

**Figure 2:**
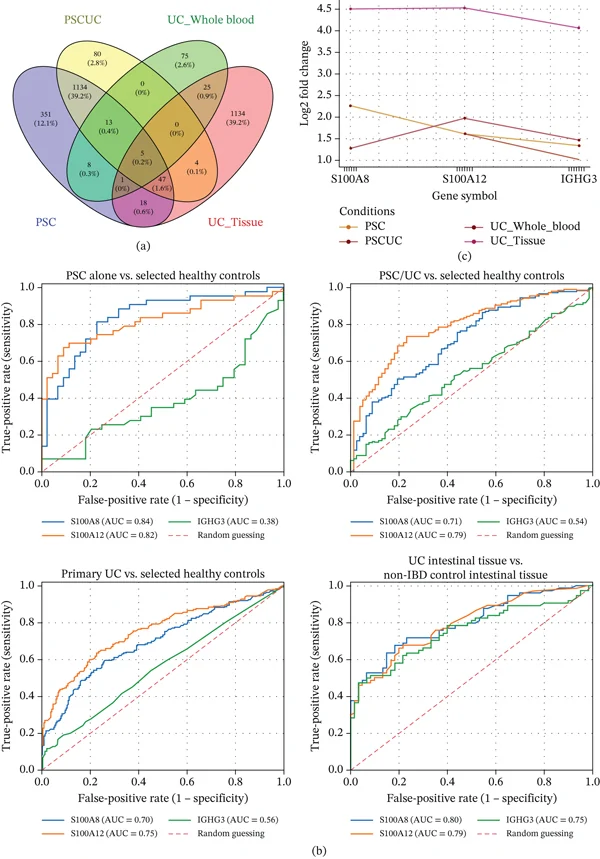
Expression overlap and discriminatory performance of candidate genes across the studied comparisons. (a) Venn diagram showing the overlap of upregulated genes identified in primary UC versus selected healthy controls in whole blood, PSC alone versus selected healthy controls in whole blood, PSC/UC versus selected healthy controls in whole blood, and UC intestinal tissue versus non‐IBD control intestinal tissue. (b) Receiver operating characteristic (ROC) curves showing the discriminatory performance of S100A8, S100A12, and IGHG3 across four disease–control comparisons. Within panel b, the ROC subplots are labeled as follows: PSC alone versus selected healthy controls; PSC/UC versus selected healthy controls; primary UC versus selected healthy controls; and UC intestinal tissue versus non‐IBD control intestinal tissue. The area under the curve (AUC) for each gene is displayed in the corresponding subplot, and the dashed diagonal line represents the performance expected from random classification. (c) Log_2_ fold changes of *S100A8*, *S100A12*, and *IGHG3* across the studied comparisons.

### 3.4. Extracellular Space Genes Identified as Potential Biomarkers

Using the DAVID database, we analyzed the common genes from the Venn diagram to identify extracellular space genes, which are more likely to be detected in blood because of their secretion or extracellular localization. This analysis identified three key extracellular space genes, *S100A12*, *S100A8*, and *IGHG3*, that warrant further investigation as noninvasive biomarkers for disease diagnosis and monitoring.

### 3.5. Discriminatory Performance of Candidate Genes Across Disease‐Control Comparisons

Receiver operating characteristic curve analysis was performed to evaluate the discriminatory performance of *S100A8*, *S100A12*, and *IGHG3* in four comparisons: PSC alone versus selected healthy controls, PSC/UC versus selected healthy controls, primary UC versus selected healthy controls, and UC intestinal tissue versus non‐IBD control intestinal tissue. In the PSC‐alone comparison, the AUC values were 0.84 for *S100A8*, 0.82 for *S100A12*, and 0.38 for *IGHG3*. In the PSC/UC comparison, the corresponding AUC values were 0.71, 0.79, and 0.54, respectively. In the primary UC comparison, the AUC values were 0.70 for *S100A8*, 0.75 for *S100A12*, and 0.56 for IGHG3. In the intestinal‐tissue comparison, *S100A8*, *S100A12*, and *IGHG3* yielded AUC values of 0.80, 0.79, and 0.75, respectively. Overall, *S100A8* and *S100A12* demonstrated more consistent discriminatory performance than *IGHG3* across the studied comparisons and were therefore retained as the principal candidate genes for subsequent analyses (Table 2 and Figure 2b).

**Table 2:** Area under the ROC curve values for *S100A8*, *S100A12*, and *IGHG3* across the four disease‐control comparisons.

Condition	Gene	AUC
(PSC alone vs. selected healthy controls)	*S100A8*	0.84
(PSC alone vs. selected healthy controls)	*S100A12*	0.82
(PSC alone vs. selected healthy controls)	*IGHG3*	0.38
(PSC/UC vs. selected healthy controls)	*S100A8*	0.71
(PSC/UC vs. selected healthy controls)	*S100A12*	0.79
(PSC/UC vs. selected healthy controls)	*IGHG3*	0.54
(Primary UC vs. selected healthy controls)	*S100A8*	0.70
(Primary UC vs. selected healthy controls)	*S100A12*	0.75
(Primary UC vs. selected healthy controls)	*IGHG3*	0.56
(UC intestinal tissue vs. non‐IBD control intestinal tissue)	*S100A8*	0.80
(UC intestinal tissue vs. non‐IBD control intestinal tissue)	*S100A12*	0.79
(UC intestinal tissue vs. non‐IBD control intestinal tissue)	*IGHG3*	0.75

### 3.6. Direct Disease–Disease Comparisons of *S100A8* and *S100A12*

To further evaluate whether S100A8 and S100A12 were associated with PSC rather than reflecting inflammatory activity related to UC alone, direct disease‐disease comparisons were performed. In the PSC/UC versus UC comparison, both genes were markedly upregulated, with log_2_ fold changes of 2.27 for *S100A8* (adjusted *p* = 3.43 × 10^−108^) and 1.61 for *S100A12* (adjusted *p* = 2.96 × 10^−65^). Similarly, in PSC alone versus UC, S100A8 showed a log_2_ fold change of 2.26 (adjusted *p* = 1.60 × 10^−31^), whereas *S100A12* showed a log_2_ fold change of 1.63 (adjusted *p* = 5.20 × 10^−17^). In contrast, direct comparison of PSC/UC with PSC alone showed no statistically significant difference for either *S100A8* (log_2_ fold change = −0.42, adjusted *p* = 0.186) or *S100A12* (log_2_ fold change = −0.30, adjusted *p* = 0.462). These findings indicate that both genes were more highly expressed in PSC‐containing groups than in UC alone, whereas concomitant UC did not produce a significant additional difference among patients with PSC. Nevertheless, because the UC‐only and PSC‐related groups originated from different source cohorts, these findings should be interpreted as exploratory rather than definitive evidence of PSC specificity (Table 3).

**Table 3:** Direct disease–disease differential‐expression comparisons of *S100A8* and *S100A12* in GSE177044.

Comparison	Gene	log2*F* *C*	*p*	Adjusted *p*
(PSC/UC vs. UC)	*S100A8*	2.27	3.50 × 10^−109^	3.43 × 10^−108^
(PSC/UC vs. UC)	*S100A12*	1.61	5.47 × 10^−66^	2.96 × 10^−65^
(PSC alone vs. UC)	*S100A8*	2.26	1.46 × 10^−32^	1.60 × 10^−31^
(PSC alone vs. UC)	*S100A12*	1.63	9.92 × 10^−18^	5.20 × 10^−17^
PSC/UC vs PSC alone	*S100A8*	−0.42	0.024	0.186
(PSC/UC vs. PSC alone)	*S100A12*	−0.30	0.160	0.462

### 3.7. Comparative Expression of Candidate Genes Across Different Conditions

The comparative analysis of differential gene expression across PSC, PSCUC, UC (whole blood), and UC (tissue) revealed distinct expression patterns for the candidate genes *S100A8*, *S100A12*, and *IGHG3*. *S100A8* exhibited higher expression in UC (whole blood) than in other conditions, with a consistent decrease observed from PSC to UC (tissue). Similarly, *S100A12* showed relatively stable expression across PSC and PSCUC, with a slight decline in PSCUC, whereas UC (whole blood) displayed elevated expression compared with UC (tissue), indicating systemic divergence. In contrast, *IGHG3* demonstrated pronounced tissue specificity, with substantially higher expression in UC (tissue) and a notable decline in PSCUC and PSC. These findings suggest that *S100A8* and *S100A12* may serve as potential biomarkers of systemic inflammation. At the same time, *IGHG3* highlights localized tissue‐specific processes in UC, emphasizing the importance of analyzing both systemic and tissue‐specific gene expression for biomarker discovery and understanding disease mechanisms in PSC, PSCUC, and UC (Figure 2c).

### 3.8. Identification of miRNA Networks Regulating Candidate Genes

The miRNet analysis revealed that *S100A8* and *S100A12* are coregulated by hsa‐mir‐20a‐5p and hsa‐mir‐671‐5p. Further functional analysis of all miRNAs in the network using the Function Explorer in the miRNet database indicated that these miRNAs are primarily involved in innate immunity, cell proliferation, cell death, cell cycle, and onco‐miRNAs. These findings highlight their potential role in regulating key BPs and pathways associated with PSC and UC pathogenesis, emphasizing their relevance as potential therapeutic and diagnostic targets (Table 4) (Figure 3a).

**Table 4:** miRNA networks regulating candidate genes involved in innate immunity, cell proliferation, cell death, cell cycle, and onco‐miRNAs, highlighting therapeutic and diagnostic potential.

Name	Hits	Adjusted *p*
Innate immunity	15	0.000117
Cell proliferation	17	0.000588
Cell death	16	0.00259
Cell cycle	15	0.00524

**Figure 3:**
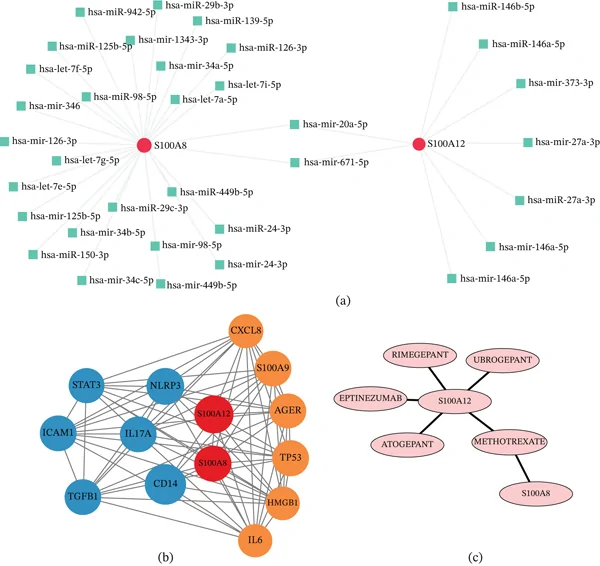
Gene interaction network. (a) miRNA‐gene interaction network illustrating the regulatory connections between candidate genes and associated miRNAs. The network highlights potential interactions relevant to disease pathogenesis and regulatory mechanisms. (b) Gene interaction network highlighting direct and indirect connections between key candidate genes and associated genes. The network illustrates potential coexpression patterns and interactions relevant to disease pathways. (c) Gene‐drug interaction network illustrating the associations between candidate genes and therapeutic compounds. The network highlights potential drug targets regulating these genes, providing insights into therapeutic strategies.

### 3.9. Coexpression Network Analysis

The coexpression network analysis revealed that *S100A8* and *S100A12* coexpress with *CXCL8*, *S100A9*, *AGER*, *TP53*, *HMGB1*, and *IL6*. Additionally, these genes exhibit indirect coexpression with *NLRP3*, *IL17A*, *CD14*, *STAT3*, *ICAM1*, and *TGFB1*. These interactions highlight the central role of *S100A8* and *S100A12* in the network, suggesting their involvement in key pathways associated with inflammation, immunity, and cellular signaling in PSC and UC pathogenesis (Figure 3b).

### 3.10. Gene‐Drug Interaction Analysis

The gene‐drug interaction analysis using the DGIdb database identified key regulatory interactions for our candidate genes. The constructed gene‐drug interaction network revealed that both *S100A8* and *S100A12* are regulated by methotrexate (MTX), a widely used therapeutic agent. These findings highlight the potential of MTX as a regulator of these genes, suggesting its relevance in the treatment or modulation of pathways associated with PSC and UC pathogenesis (Figure 3c).

### 3.11. Integration, Clustering, and Monocyte Analysis Across Disease States

A total of 16 scRNA‐seq datasets were collected from the GEO database (GSE247128 and GSE231993), including four normal colon, seven UC, three PSC, and two PSC with prior UC samples. After removing cells expressing fewer than three genes or with mitochondrial gene content above 20%, each sample underwent an outlier filter based on median ± 2SD of nFeature_RNA. As summarized in Table S2, the number of retained cells per sample ranged from approximately 565 to 17,006, reflecting variations in initial library complexity and sequencing depth. Following quality control, samples from each disease group were integrated separately via Seurat′s rPCA workflow. The resulting integrated objects were defined in four central states normal colon, UC, PSC, and PSC with UC and subsequently scaled, clustered, and visualized with UMAP (Figure S1). For future analysis, four integrated samples were merged, scaled, and visualized by UMAP. This showed clear segregation by disease state, with PSC and PSC with UC occupying distinct yet partially overlapping transcriptomic spaces, whereas UC and normal colon formed separate clusters (Figure 4a). Two candidate marker genes, *S100A8* and *S100A12*, were examined within the monocyte compartment. FeaturePlots split by each state indicated higher expression of these genes in PSC and PSC with UC monocytes compared with UC and normal colon (Figure 4b). RidgePlots further supported these distributional differences, demonstrating elevated expression in PSC‐related groups relative to UC and normal colon. (Figure 4c). Because of varying monocyte counts across disease groups, a harmonization formula was applied to standardize the number of marker positive cells to a hypothetical population of 100,000 monocytes. As illustrated in the scatter plot (Figure 4d), the proportion of *S100A8*‐positive and *S100A12*‐positive monocytes increased dramatically from normal colon to UC, and reached maximal levels in PSC with UC and PSC. *S100A12* showed the highest relative increase, suggesting it could be a potential biomarker for PSC‐associated disease activity. List of gene markers used for cluster annotation is presented in Table S3.

**Figure 4:**
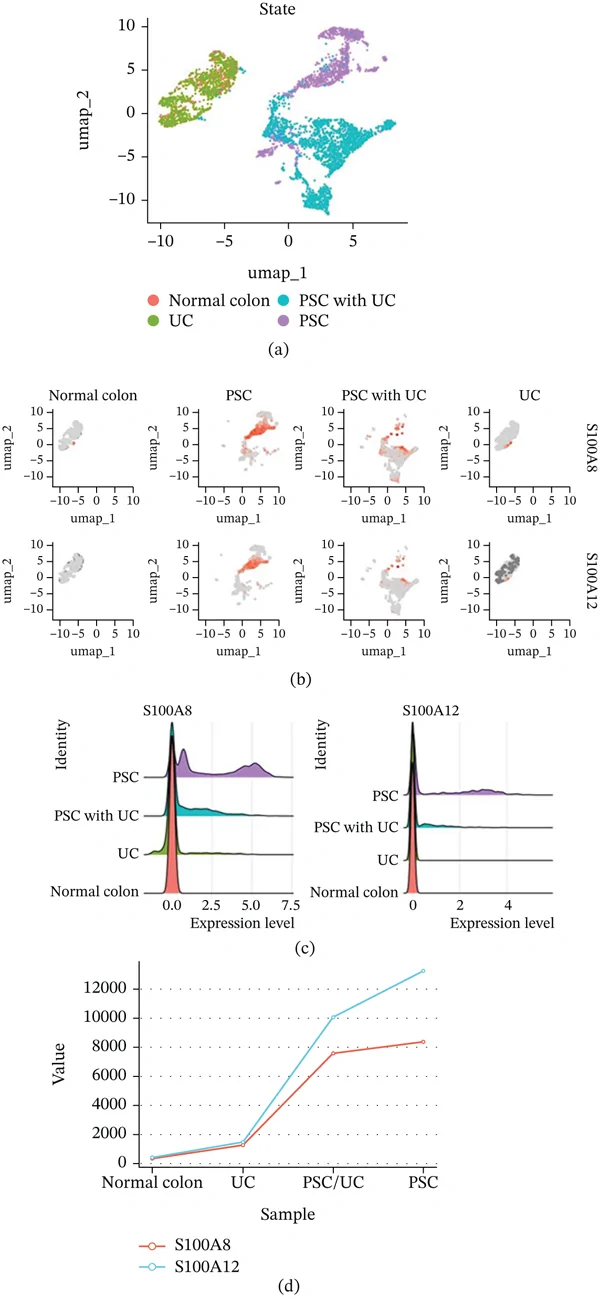
UMAP, density and line plots. (a) UMAP visualization of merged samples, segregated by states of disease. Color‐coded based on sample origin: normal colon (red), ulcerative colitis (UC, green), primary sclerosing cholangitis (PSC, purple), and PSC with prior UC history (cyan). (b) UMAP feature plots showing the expression patterns of *S100A8* (top row) and *S100A12* (bottom row) across different sample groups. (c) Density plots showing the expression levels of *S100A8* (left) and *S100A12* (right) across different sample groups. (d) Line plot depicting *S100A8* (red) expression levels and *S100A12* (blue) across different sample groups.

## 4. Discussion

Despite the low prevalence of PSC, its diagnosis remains critically important in patients with IBD, particularly UC. Although the incidence of PSCUC is relatively low (1.67%), a significant proportion (60%–80%) of PSC patients have concomitant IBD, predominantly UC [5, 18, 19]. Although the global prevalence of IBD is increasing, the incidence of PSC‐IBD is rising even more rapidly, suggesting that PSC‐IBD may represent a distinct phenotype within the IBD spectrum [20]. Patients with PSC‐IBD often exhibit unique clinical features that differentiate them from those with IBD alone. Moreover, PSC‐IBD is associated with a higher risk of colorectal and hepatobiliary malignancies than IBD without PSC [21]. Therefore, early identification of PSC‐IBD in patients already diagnosed with IBD is essential to mitigate disease progression and associated complications. In this study, we aimed at identifying new biomarkers for earlier diagnosis of PSCUC. The study identified distinct and overlapping DEGs across UC, PSC, and PSCUC in blood and tissue samples. *S100A8* and *S100A12* emerged as potential noninvasive biomarkers with substantial diagnostic value for PSC‐IBD. In addition, scRNA‐seq analysis supported increased expression of *S100A8* and *S100A12* in monocytes from PSC‐related groups. The miRNet analysis identified hsa‐mir‐20a‐5p and hsa‐mir‐671‐5p as key regulators of *S100A8* and *S100A12*. *S100A8* and *S100A12* are coexpressed with *CXCL8*, *S100A9*, *TP53*, *HMGB1*, and IL6, emphasizing their role in inflammatory and immune responses

To further examine whether the observed expression patterns reflected PSC‐associated biology rather than generalized UC‐related inflammation, we performed direct disease–disease comparisons. Both *S100A8* and *S100A12* were markedly upregulated in PSC/UC relative to UC alone and in PSC alone relative to UC, whereas no significant differences were observed for either gene between PSC/UC or PSC alone after multiple‐testing correction. This pattern suggests that increased expression of these genes may be more closely associated with the presence of PSC than with the coexistence of UC. Importantly, however, the UC‐only and PSC‐related samples in GSE177044 originated from different source cohorts, and residual cohort‐related demographic or technical effects cannot be excluded. Therefore, these results strengthen the evidence for a PSC‐associated expression pattern but should not yet be interpreted as definitive proof of PSC‐specific diagnostic performance. Independent validation in a cohort containing UC‐only, PSC‐alone, and PSC/UC patients recruited and processed under the same conditions is warranted.

*S100A8* and *S100A12* belong to the S100 protein family, a group of acidic calcium‐binding proteins. They exhibit a high affinity for calcium ions (Ca^2+^) and play crucial roles in various intracellular and extracellular functions through calcium‐dependent interactions [22]. *S100A8*, alongside S100A12, is highly expressed in myeloid cells and plays a crucial role in inflammatory responses. They participate in immune regulation by interacting with receptors such as toll‐like receptor 4 (TLR4) and the receptor for advanced glycation end products (RAGE), which leads to cytokine production and immune cell activation [23]. Calprotectin, a clinically established marker of intestinal inflammation, is a heterodimer composed of S100A8 and S100A9; thus, S100A8 encodes one of its two protein components, whereas *S100A12* is a related but distinct S100‐family inflammatory protein [13, 14]. In addition, fecal calprotectin has been investigated as a potential noninvasive marker of intestinal inflammation and as a surrogate marker of biliary inflammation in patients with PSC‐IBD [24]. This provides relevant clinical context for the present identification of S100A8, although our transcriptomic analysis does not directly assess fecal calprotectin concentrations or establish its diagnostic utility for PSC in UC. Human *S100A8*/*S100A9* is chemotactic for neutrophils and influences the migration of other cell types, including myeloid‐derived suppressor cells and some tumor cells, and may facilitate tumor cell invasion [25]. *S100A12* is a multifunctional protein primarily involved in immune regulation, inflammation, antimicrobial defense, and cancer prognosis. It also has emerging roles in neurological aging and skin diseases, making it a potential biomarker for various inflammatory and pathological conditions [26–29].

Our coexpression network analysis identified *S100A8* and *S100A12* as central hub genes, exhibiting direct coexpression with key inflammatory and immune‐related genes, including *CXCL8*, *S100A9*, *AGER*, *TP53*, *HMGB1*, and *IL6*. These genes showed indirect coexpression with critical mediators, including *NLRP3*, *IL17A*, *CD14*, *STAT3*, *ICAM1*, and *TGFB1*, further highlighting their involvement in immune regulation and inflammation. These interactions suggest that *S100A8* and *S100A12* are potential biomarkers for PSC‐IBD and may also play an active role in disease pathogenesis.

*S100A8* and *S100A12*, both members of the S100 protein family, are known for their roles in calcium signaling, immune modulation, and inflammatory responses. Their coexpression with CXCL8 (IL‐8), a potent neutrophil chemoattractant, underscores their role in immune cell recruitment and persistent inflammation in PSC‐IBD. CXCL8 is a key driver of neutrophil infiltration in the intestinal mucosa, contributing to tissue damage and exacerbating the chronic inflammatory response in IBD [30]. Elevated CXCL8 levels have been reported in UC and Crohn′s disease, correlating with disease severity and mucosal damage [31]. The interplay between CXCL8 and *S100A8*/*S100A12* suggests a feed‐forward loop that amplifies inflammation in PSC‐IBD, potentially promoting disease progression.

Furthermore, the indirect coexpression of *S100A8* and *S100A12* with NLRP3, a key component of the NLRP3 inflammasome, reinforces their involvement in innate immune activation and epithelial damage. The NLRP3 inflammasome serves as a crucial sensor of cellular stress and danger signals, promoting caspase‐1 activation and the release of pro‐inflammatory cytokines IL‐1*β* and IL‐18 [32]. In IBD, dysregulated NLRP3 activation has been implicated in epithelial barrier dysfunction, immune hyperactivation, and fibrosis, further exacerbating intestinal inflammation [33] The observed association between S100A8/S100A12 and NLRP3 suggests a mechanistic link. S100 proteins may function as danger‐associated molecular patterns (DAMPs), triggering inflammasome activation and sustaining chronic inflammation in PSC‐IBD.

However, conflicting evidence exists regarding the role of NLRP3 in inflammation, as studies in OVA‐BIL mice have shown that NLRP3 deficiency leads to an uncontrolled disease course upon the induction of a cholangiocyte‐specific immune response [34]. This paradox suggests that NLRP3 may have pathogenic and protective roles, depending on the disease context. Therefore, further investigation is required to delineate the precise function of NLRP3 in PSC‐IBD pathogenesis, particularly its interplay with *S100A8*/*S100A12* and its dual role in inflammation regulation. The association with TP53 and HMGB1, both involved in cellular stress responses and tissue remodeling, suggests a potential link between chronic inflammation and epithelial damage or fibrosis in PSC‐IBD patients.

Moreover, the indirect coexpression with NLRP3 and IL17A, central components of inflammasome activation and Th17‐driven immunity, further supports the hypothesis that *S100A8* and *S100A12* contribute to immune dysregulation in PSC‐IBD. Given that Th17 signaling is implicated in both IBD and PSC, the interplay between these pathways may be crucial in the development of the PSC‐IBD phenotype [35].

The IL6‐STAT3 axis, another key component of the coexpression network, plays a fundamental role in immune cell differentiation, cytokine signaling, and chronic inflammation [36]. Elevated IL‐6 levels in PSC‐IBD drive STAT3 activation, which promotes the expansion of pathogenic Th17 cells and suppresses regulatory T cells (Tregs), exacerbating mucosal inflammation and immune dysregulation [37]. The involvement of *S100A8*/*S100A12* in this axis suggests a role in sustaining IL‐6‐driven immune responses, thereby leading to persistent inflammation and potentially progressing to fibrosis. A recent study reported that increased IL‐6‐dependent activation of transcription factor STAT3 in PSC leads to enhanced production of IFN gamma and IL‐17A in CD4+ T cells and increased systemic inflammation [38].

Overall, the coexpression network analysis demonstrates that *S100A8* and *S100A12* are highly interconnected with key inflammatory, immune‐regulatory, and fibrotic pathways, reinforcing their potential as biomarkers for PSC‐IBD. Their association with *CXCL8*, *NLRP3*, *AGER*, *IL6*, *STAT3*, *ICAM1*, and *TGFB1* suggests that these genes collectively contribute to immune cell recruitment, inflammasome activation, chronic inflammation, and fibrosis progression, all of which are central to PSC‐IBD pathogenesis. These findings highlight the importance of targeting these molecular pathways for therapeutic interventions and support the potential of *S100A8* and *S100A12* as biomarkers for early disease detection and progression monitoring.

In our analysis, hsa‐miR‐20a‐5p and hsa‐miR‐671‐5p were identified as key microRNAs (miRNAs) coregulating *S100A8* and *S100A12*, suggesting their potential role in modulating inflammation and immune responses in PSC‐IBD. hsa‐miR‐20a‐5p, a member of the miR‐17‐92 cluster, is known for its role in immune regulation, primarily by targeting pathways involved in NF‐*κ*B signaling, T‐cell differentiation, and cytokine production [39]. Its dysregulation has been linked to various inflammatory diseases, where it may function as a pro‐inflammatory or anti‐inflammatory modulator, depending on the cellular context. Interestingly, an experimental study found that the miR‐20a‐5p/TGFBR2 axis is a key regulator of TGF‐*β* signaling, underscoring the critical role of miR‐20a‐5p in liver fibrosis development. In the study above, a lower level of miR‐20a‐5p exacerbates inflammation, whereas its upregulation suppresses cytokine release [40]. hsa‐miR‐671‐5p has been associated with the post‐transcriptional regulation of immune and inflammatory mediators, including those involved in fibrosis, epithelial‐mesenchymal transition (EMT), and macrophage polarization [41]. The interactions of miR‐671 have been widely explored in both malignant and nonmalignant conditions. miR‐671‐5p has tumor suppressing and oncogenic roles depending on the cancer type and biological context. It influences key pathways related to cell proliferation, migration, and invasion, like SMAD3 (37), FOXM1 (38), and the Wnt signaling pathway [42]. Although miR‐671‐5p has been reported to be upregulated in advanced fibrosis in patients with chronic hepatitis B, the evidence regarding its specific role in PSC‐associated fibrogenesis remains indirect. Further studies in PSC‐specific cohorts are needed to determine whether miR‐671‐5p contributes to fibrogenesis in PSC [43]. The coregulation of *S100A8* and *S100A12* by these miRNAs suggests a possible posttranscriptional control mechanism influencing innate immune activation, neutrophil recruitment, and inflammasome signaling, all of which play critical roles in PSC‐IBD pathogenesis.

Our gene‐drug interaction analysis identified *S100A8* and *S100A12* as targets of MTX, a widely used immunosuppressive and anti‐inflammatory agent. MTX, commonly prescribed for autoimmune diseases, exerts its effects by inhibiting dihydrofolate reductase (DHFR), leading to reduced DNA synthesis, suppression of T‐cell activation, and decreased production of pro‐inflammatory cytokines [44]. Recently, MTX has gained attention as a therapeutic option for IBD, particularly in Crohn′s disease, because of concerns over thiopurine‐associated malignancies. Consequently, MTX has re‐emerged as a potential alternative therapy for IBD [45]. In the 1990s, MTX was explored as a treatment for PSC, but early‐phase trials yielded inconclusive results, primarily because of small sample sizes, short study durations, and heterogeneous patient populations [46]. Given these limitations, the role of MTX in PSC‐IBD remains an open question, warranting further investigation into its potential disease‐modifying effects in this subset of patients.

Our scRNA‐seq analysis provided crucial insights into the expression patterns of *S100A8* and *S100A12* across different disease states. Notably, both genes exhibited significantly higher expression in monocytes from PSCUC patients than those from UC‐only and standard controls. Feature and ridge plot analyses further supported this elevated expression, highlighting a progressive increase from normal colon to UC, with peak levels observed in PSCUC monocytes. This suggests a potential role for *S100A8* and *S100A12* as key mediators of monocyte‐driven inflammation in PSCUC, reinforcing their involvement in the disease′s characteristic innate immune response and chronic inflammation.

This study has several limitations. First, the findings were derived from publicly available transcriptomic datasets and in silico analyses, so there are no experimentally validated using independent biological samples. Validation by RT‐qPCR, ELISA, flow cytometry, or other protein level assays in an independent clinical cohort would strengthen the evidence for *S100A8* and *S100A12* as candidate biomarkers. Second, heterogeneity among the source datasets, including differences in cohort composition, sample type, and potentially other clinical or technical factors, may limit the reproducibility and generalizability of the findings. Third, the scRNA‐seq analysis was based on a limited number of biological samples (16 samples in total), including only two PSC samples with prior UC. Therefore, the observed cell‐type‐specific expression patterns of *S100A8* and *S100A12* should be considered exploratory and supportive of the bulk transcriptomic findings rather than definitive validation. Larger independent single‐cell cohorts, particularly those including more patients with PSC/UC, are required to confirm the reproducibility and generalizability of these observations. Fourth, the present findings demonstrate associations but do not establish a causal role for *S100A8* and *S100A12* in PSC‐IBD pathogenesis; functional studies involving gene knockdown, overexpression, or other mechanistic approaches would be required to investigate causality. Finally, the accession identifiers of all selected GSE177044 healthy‐control samples and the selected UC subsets used in the direct disease‐disease comparisons were not retained. Consequently, demographic characteristics could not be reconstructed specifically for these selected analytical subsets. In addition, the comparisons were not adjusted for age, sex, treatment exposure, disease duration, or other potential covariates; therefore, residual confounding, cohort‐related effects, and selection‐related variability cannot be excluded.

In conclusion, *S100A8* and *S100A12* were identified as candidate blood‐accessible biomarkers for distinguishing PSC‐related disease, with scRNA‐seq analysis supporting their increased expression in monocytes from PSC and PSC/UC samples. These findings suggest that both genes reflect myeloid‐associated inflammatory activity; however, their diagnostic specificity and clinical utility remain to be confirmed. Independent prospective studies using RT‐qPCR and protein‐level assays are required before these candidates can be considered for clinical application.

**Nomenclature**
S100A8 and S100A12: Calcium‐binding proteins of the S100 family, predominantly expressed in myeloid cells and strongly associated with inflammatory responses, innate immune activation, and neutrophil recruitment.
area under the curve (AUC): A quantitative measure of a ROC curve that indicates the diagnostic accuracy of a test or biomarker (values closer to 1.0 represent better performance).
calprotectin (S100A8/S100A9 complex): A heterodimeric complex widely used as a clinical biomarker for intestinal inflammation, particularly in inflammatory bowel disease.
cholestasis: Impaired bile flow resulting in the accumulation of bile acids in the liver and bloodstream.
differentially expressed genes (DEGs): Genes that show statistically significant differences in expression levels between two or more experimental conditions.
extracellular space genes: Genes encoding proteins that are secreted or located outside cells, making them detectable in body fluids such as blood.
FeaturePlot and RidgePlot: Visualization tools in Seurat used for displaying gene expression patterns across cell populations.
Gene Expression Omnibus (GEO): A public repository of high‐throughput functional genomic data, including RNA‐Seq datasets.
inflammasome (NLRP3): A multiprotein intracellular complex that activates inflammatory caspases, leading to IL‐1*β* and IL‐18 production and pyroptosis.
log2 fold change (log2FC): A measure indicating how many times a gene’s expression changes between two groups, converted to a logarithmic scale (base 2).
primary sclerosing cholangitis (PSC): A chronic, progressive cholestatic liver disease characterized by inflammation, fibrosis, and stricturing of intrahepatic and/or extrahepatic bile ducts, often associated with inflammatory bowel disease.
PSC‐associated ulcerative colitis (PSCUC): A distinct phenotype in which patients have both PSC and UC, often with atypical inflammatory patterns and a higher risk of colorectal and hepatobiliary cancers.
receiver operating characteristic (ROC) curve: A diagnostic tool used to evaluate the performance of a biomarker by plotting sensitivity versus specificity.
Receptor for Advanced Glycation End Products (RAGE): A pattern recognition receptor recognizing inflammatory ligands such as S100 proteins, amplifying immune responses.
reciprocal PCA (rPCA) integration: A method in Seurat for integrating multiple scRNA‐seq datasets while minimizing batch effects.
shared nearest neighbor (SNN) clustering: A graph‐based clustering method that groups cells based on similarity in transcriptomic profiles.
single‐cell RNA sequencing (scRNA‐seq): A high‐resolution transcriptomic technique that profiles gene expression of individual cells, enabling characterization of cell populations and states.
ulcerative colitis (UC): A chronic inflammatory bowel disease characterized by continuous mucosal inflammation of the colon and rectum, leading to ulceration and gastrointestinal symptoms.
Uniform Manifold Approximation and Projection (UMAP): A dimensionality reduction technique used to visualize high‐dimensional data (e.g., scRNA‐seq) in two dimensions.

## Author Contributions

Pedram Asadi Sarabi and Alireza Beheshti‐Maal: conceptualization, investigation, methodology, formal analysis, visualization, writing‐original draft. Amir Ali Judaki: methodology, formal analysis, visualization, writing‐original draft. Seyedeh Kiana Teymoorian: investigation, writing‐original draft. Zohreh Hashemian: investigation, visualization, writing‐original draft. Elham Rismani: visualization, writing‐review and editing. Asma Gul, Reza Malekzadeh, and Moustapha Hassan: writing‐review and editing. Massoud Vosough: conceptualization, supervision, validation, writing‐review and editing. Pedram Asadi Sarabi and Alireza Beheshti‐Maal contributed equally to this work.

## Funding

No funding was received for this manuscript.

## Disclosure

All authors approved the final manuscript.

## Conflicts of Interest

The authors declare no conflicts of interest.

## Supporting information


**Supporting Information** Additional supporting information can be found online in the Supporting Information section. Figure S1: UMAP visualization of major cell populations across study groups. (a) Normal colon: Uniform Manifold Approximation and Projection (UMAP) plot showing the distribution of major cell types identified in healthy colon samples, including T cells, epithelial cells, plasma cells, B cells, fibroblasts, monocytes, endothelial cells, mast cells, proliferating cells, and glial cells. (b) PSC: UMAP embedding of single‐cell transcriptomes from patients with primary sclerosing cholangitis (PSC), illustrating distinct clusters corresponding to NK cells, B‐cell subsets, T cells, monocytes, hepatocyte subpopulations, liver sinusoidal endothelial cells (LSECs), plasma cells, and proliferating cells. (c) PSCUC: integrated UMAP projection of samples from patients with primary sclerosing cholangitis associated with ulcerative colitis (PSCUC), demonstrating major immune and stromal populations including T cells, Kupffer cell precursors, LSECs, monocytes, Kupffer cells, hepatocytes, fibroblast cells, and B cells. (d) UC: UMAP representation of cell populations in Ulcerative Colitis (UC) samples, showing clustering of T cells, epithelial cells, plasma cells, B cells, fibroblasts, monocytes, endothelial and mast cells, proliferating cells, and glial cells. Table S1: Available demographic and clinical characteristics of the source cohorts represented in the bulk RNA sequencing datasets. Table S2: Quality control and cluster marker identification across scRNA‐seq datasets. It provides details on datasets including sample ID, minimum and maximum nFeature, and the number of cells before and after QC. Table S3: List of gene markers used for cluster annotation. It provides list of gene markers in epithelial cells, T cells, plasma cells, B cells, fibroblasts, monocytes, endothelial cells, proliferating cells, glial cells or Schwann cells, mast cells, NK cells, hepatocytes, and liver sinusoidal endothelial cells (LSECs).

## Data Availability

The datasets analyzed in this study are publicly available in the NCBI Gene Expression Omnibus under accession numbers GSE177044, GSE83687, GSE247128, and GSE231993.
