# Supplementary material for: Noninvasive Biomarkers for Early Detection of Primary Sclerosing Cholangitis in Ulcerative Colitis
Source: Biomed Res Int. 2026 Oct 17;2026:9547868. doi: 10.1155/bmri/9547868 (PMC13675833; doi:10.1155/bmri/9547868)
Supplement: Supplementary file 1 [file BMRI-2026-9547868-s001.docx]

Table S1. Available demographic and clinical characteristics of the source cohorts represented in the bulk RNA-sequencing datasets

| **Dataset and group** | **Total, n** | **Age available, n** | **Age, mean ± SD, years** | **Age, median (IQR), years** | **Male, n (%)** | **Female, n (%)** | **Years since PSC diagnosis** |
| --- | --- | --- | --- | --- | --- | --- | --- |
| GSE83687: UC tissue | 32 | 32 | 39.2 ± 20.5 | 32.0 (24.0–54.0) | 20 (62.5) | 12 (37.5) | Not applicable |
| GSE83687: non-IBD control tissue | 60 | 60 | 62.5 ± 16.8 | 64.0 (51.8–73.0) | 25 (41.7) | 35 (58.3) | Not applicable |
| GSE177044: primary UC, German source cohort | 495 | 301 | 41.0 ± 14.2 | 41.0 (31.0–51.0) | 99/246 (40.2) | 147/246 (59.8) | Not applicable |
| GSE177044: healthy controls, German source cohort | 243 | 243 | 49.8 ± 12.6 | 49.0 (40.0–60.0) | Not available | Not available | Not applicable |
| GSE177044: PSC alone, Norwegian source cohort | 43 | 43 | 48.0 ± 13.1 | 47.0 (37.5–58.5) | 25 (58.1) | 18 (41.9) | 7.8 ± 8.1; available for 40/43 |
| GSE177044: PSC/UC, Norwegian source cohort | 177 | 177 | 48.4 ± 13.8 | 49.0 (39.0–58.0) | 140 (79.1) | 37 (20.9) | 12.3 ± 9.7; available for 174/177 |
| GSE177044: healthy controls, Norwegian source cohort | 77 | 77 | 41.7 ± 6.9 | 40.0 (36.0–45.0) | 51 (66.2) | 26 (33.8) | Not applicable |

**Note:** Values were calculated from publicly available GEO metadata and describe the complete source cohorts represented in GSE177044 and GSE83687. For GSE177044, the demographic values shown do not necessarily represent the exact selected analytical subsets because the accession identifiers of all selected control samples were not retained and subset-specific demographic characteristics could not be reconstructed. Samples were not individually or frequency matched according to age, sex, treatment status, disease duration, or other demographic or clinical characteristics. Percentages were calculated among samples with available information, and missing values were not imputed. **n**: Number.

Table S2: Quality Control and Cluster Marker Identification Across scRNA-seq Datasets.

| **Dataset** | **Type** | **Sample ID** | **Min Cell** | **Min nFeature** | **Max nFeature** | **MT percentage** | **No. cell before QC** | **No. cell after QC** |
| --- | --- | --- | --- | --- | --- | --- | --- | --- |
| **GSE247128** | PSC | GSM7883942 | 3 | 0 | 3561 | 20 | 7351 | 2811 |
|  |  | GSM7883944 | 3 | 217 | 2387 | 20 | 8555 | 8024 |
|  |  | GSM7883946 | 3 | 0 | 2834 | 20 | 843 | 777 |
|  | PSC/UC | GSM7883940 | 3 | 0 | 3240 | 20 | 2898 | 1960 |
|  |  | GSM7883941 | 3 | 0 | 4575 | 20 | 22076 | 17006 |
| **GSE231993** | Normal UC | GSM7307094 | 3 | 0 | 2952 | 20 | 4124 | 2355 |
|  |  | GSM7307095 | 3 | 0 | 2506 | 20 | 5383 | 4327 |
|  |  | GSM7307096 | 3 | 0 | 2723 | 20 | 3557 | 1978 |
|  |  | GSM7307097 | 3 | 0 | 2648 | 20 | 5765 | 4259 |
|  | UC | GSM7307099 | 3 | 0 | 2189 | 20 | 5480 | 4963 |
|  |  | GSM7307100 | 3 | 0 | 2942 | 20 | 4022 | 3005 |
|  |  | GSM7307101 | 3 | 0 | 2605 | 20 | 4790 | 3971 |
|  |  | GSM7307102 | 3 | 0 | 1397 | 20 | 4781 | 3728 |
|  |  | GSM7307103 | 3 | 0 | 1934 | 20 | 4083 | 2881 |
|  |  | GSM7307104 | 3 | 0 | 2609 | 20 | 5841 | 3817 |
|  |  | GSM7307105 | 3 | 0 | 2880 | 20 | 5660 | 4072 |

Table S3: List of gene markers used for cluster annotation

| **Cluster** | **Marker** |
| --- | --- |
| **Epithelial Cells** | EPCAM, KRT8, KRT18, CD24 |
| **T Cells** | CD3D, CD3E, CD8, |
| **Plasma Cells** | SDC1, MZB1, XBP1 |
| **B Cells** | MS4A1, CD79B |
| **Fibroblasts** | VIM, FAP |
| **Monocytes** | HLA-DRA, CD14 |
| **Endothelial Cells** | CDH5, VWF, CD34, |
| **Proliferating Cells** | PCLAF, STMN1, CKS2, PCNA |
| **Glial Cells or Schwann cells** | MPZ, PMP22, PLP1, CRYAB |
| **Mast Cells** | MS4A2, CTSG, CPA3, HPGDS |
| **NK Cells** | KLRF1, FGFBP2, NKG7, NCR3 |
| **Hepatocytes** | GSTA1, CYP2E1, APOA1, ASGR1 |
| **Liver Sinusoidal Endothelial Cells (LSECs)** | CLEC4G, MRC1, LYVE1, STAB1, STAB2 |

**Figure S1. UMAP visualization of major cell populations across study groups.**
a: **Normal Colon:** Uniform Manifold Approximation and Projection (UMAP) plot showing the distribution of major cell types identified in healthy colon samples, including T cells, epithelial cells, plasma cells, B cells, fibroblasts, monocytes, endothelial cells, mast cells, proliferating cells, and glial cells. b: **PSC:** UMAP embedding of single-cell transcriptomes from patients with **Primary Sclerosing Cholangitis (PSC),** illustrating distinct clusters corresponding to NK cells, B-cell subsets, T cells, monocytes, hepatocyte subpopulations, liver sinusoidal endothelial cells (LSECs), plasma cells, and proliferating cells. c: **PSCUC:** Integrated UMAP projection of samples from patients with **Primary Sclerosing Cholangitis associated with Ulcerative Colitis (PSCUC)**, demonstrating major immune and stromal populations including T cells, Kupffer cell precursors, LSECs, monocytes, Kupffer cells, hepatocytes, fibroblast cells, and B cells. d: **UC:** UMAP representation of cell populations in **Ulcerative Colitis (UC)** samples, showing clustering of T cells, epithelial cells, plasma cells, B cells, fibroblasts, monocytes, endothelial and mast cells, proliferating cells, and glial cells.
